# [Retracted] Sp1-CD147 positive feedback loop promotes the invasion ability of ovarian cancer

**DOI:** 10.3892/or.2025.9030

**Published:** 2025-11-25

**Authors:** Jing Zhao, Wei Ye, Juan Wu, Lijuan Liu, Lina Yang, Lu Gao, Biliang Chen, Fanglin Zhang, Hong Yang, Yu Li

Oncol Rep 34: 67–76, 2015; DOI: 10.3892/or.2015.3999

Subsequently to the publication of the above paper, a concerned reader has drawn to the Editor's attention that, for the western blot data shown in Fig. 2A and B on p. 71, certain of the protein bands looked strikingly similar to others, where the results of differently performed experiments were intended to have been represented (considering both the forward orientations of the bands in question, and occasionally, bands positioned in the reverse orientation).

The Editorial Office has investigated this matter independently, and reached the same concluson as the reader that certain of the data appeared to have been duplicated within Fig. 2A and B. Given that this issue has come to light, the Editor of *Oncology Reports* has decided that this paper should be retracted from the Journal on account of a lack of confidence in the presented data. The authors were asked for an explanation to account for these concerns, but the Editorial Office did not receive a reply. The Editor apologizes to the readership for any inconvenience caused.

